# Classical biological control of the African citrus psyllid *Trioza erytreae*, a major threat to the European citrus industry

**DOI:** 10.1038/s41598-019-45294-w

**Published:** 2019-07-01

**Authors:** J. Pérez-Rodríguez, K. Krüger, M. Pérez-Hedo, O. Ruíz-Rivero, A. Urbaneja, A. Tena

**Affiliations:** 1 Centro de Protección Vegetal y Biotecnología, Instituto Valenciano de Investigaciones Agrarias (IVIA), Carretera Moncada-Náquera km 4.5, 46113 Moncada, Valencia Spain; 2 Laboratori d’Investigació d’Entomologia, Departament de Zoologia, Facultat de Ciències Biològiques, Carrer Doctor Moliner s/n, 46100 Burjassot, València Spain; 3 Department of Zoology and Entomology, University of Pretoria, Private Bag X20, Pretoria, 0028 South Africa

**Keywords:** Agroecology, Entomology, Invasive species

## Abstract

Citrus greening or huanglongbing (HLB) is the main threat to the European citrus industry since one of its vectors, the African citrus psyllid, *Trioza erytreae*, has recently become established in mainland Europe. In this context, classical biological control programmes should be implemented to reduce the spread of the psyllid. The aims of this study were to: i) disentangle the parasitoid complex of *T*. *erytreae* combining morphological and molecular characterization; and ii) to study the biology of its main parasitoids in its area of origin in South Africa for their future importation into Europe. The main citrus producing areas of South Africa were surveyed during 2017. In contrast to previous studies, the parasitoid complex of *T*. *erytreae* included three species of primary parasitoids: *Tamarixia dryi*, *Psyllaephagus pulvinatus* and another parasitoid of the genus *Tamarixia*. Molecular analysis showed that it is a new species closely related to *T*. *dryi*. *Tamarixia dryi* was the most abundant parasitoid but its relative abundance varied among sampling sites. The sex ratio (males/females) of *T*. *dryi* and *Tamarixia* sp. decreased with *T*. *erytreae* size and became female biased when psyllid nymphs were larger than 0.6 and 1.2 mm^2^, respectively. These parasitoids were attacked by three species of hyperparasitoids, *Aphidencyrtus cassatus*, *Marietta javensis* and a species of the genus *Aphanogmus*. *Aphidencyrtus cassatus*, the most abundant hyperparasitoid, tended to emerge from large nymphs, and adult females lived as long as those of *T*. *dryi*. The implications of these results are discussed within the framework of the introduction of *T*. *dryi* into Europe.

## Introduction

Citrus greening or huanglongbing (HLB) is one of the most devastating citrus diseases in the world^1–4^. It is associated with the three phloem α-proteobacterias “*Candidatus* Liberibacter asiaticus” (CLas), “*Ca*. Liberibacter americanus” (CLam) and “*Ca*. Liberibacter africanus” (CLaf). The three bacteria are transmitted by two insect vectors: the Asian citrus psyllid *Diaphorina citri* Kuwayama (Hemiptera: Liviidae) and the African citrus psyllid *Trioza erytreae* (Del Guercio) (Hemiptera: Triozidae)^2,5^. Since its first record in Taiwan in 1908^6^, *D*. *citri* has spread and has been reported transmitting CLas in Asia, parts of North and South America, Africa and numerous islands in the Atlantic and Pacific oceans^2,7–10^. In contrast, *T*. *erytreae* is associated with CLaf and, since its first record in 1929 in South Africa^11,12^, has been recorded along all the African continent, Yemen and a few Atlantic Ocean islands^2^. It has recently been reported from Portugal and Spain^13^ even though HLB has not been detected yet in European countries^14,15^.

HLB disease manifests as asymmetrical yellow mottles or severe chlorosis of the foliage, fruit drop and dieback^2,16^, leading to significant economic losses^17,18^. As an example of the destructive potential of HLB, citrus production in Florida has dropped by more than 70% since HLB was detected in 2005^19^. In the Mediterranean Basin, HLB detection could be a critical turning point not only because it is the main producing area of citrus for the fresh market in the World^20^, but also because Mediterranean citriculture is based on small farming, where managing HLB and its vectors is more complex than in larger commercial orchards^21^.

In Europe, *T*. *erytreae* was first detected in 1994 in Madeira (Portugal)^22^ and later on in the Canary Islands (Spain) in 2002^23,24^. Until then, it seemed to be restricted to non-continental areas, but in 2014 it first appeared in the north-western Iberian Peninsula^13^. Despite the initial insecticide treatments to eradicate it, *T*. *erytreae* is now spreading from the north-west to the south-west of the Iberian Peninsula^15^. Parasitoids of the genus *Tamarixia* are amongst the most effective natural enemies of HLB vectors^5,25–27^. However, no native parasitoids have been recorded from *T*. *erytreae* in the Atlantic islands or in the Iberian Peninsula^15,28^. In this context, classical biological control seems to be the most feasible measure for preventing *T*. *erytreae* to spread further in the Mediterranean citrus growing areas.

The complex of parasitoids in South Africa and Swaziland was analyzed in detail during the 1960’s and 70 s^29,30^ and in Cameroon twenty years ago^31^. According to these studies, the two main primary parasitoids of *T*. *erytreae* in Southern Africa are *Tamarixia dryi* (synonym *Tetrastichus dryi*) (Waterston) (Hymenoptera: Eulophidae) and *Psyllaephagus pulvinatus* (Waterston) (Hymenoptera: Encyrtidae). Both are solitary koinobiont parasitoids. The former is an ectoparasitoid, whereas the encyrtid is an endoparasitoid. These primary parasitoids are frequently attacked by a complex of hyperparasitoids^26,32^ that, accordingly to Van Der Berg and Greenland^26^, severely decrease the impact of the primary parasitoids. *Tamarixia dryi* was used in a classical biological control programme in Reunion Island when *T*. *erytreae* was detected in 1974^25,33^. In less than five years, *T*. *dryi* became established and controlled *T*. *erytreae*^25,26^. Similarly, in 1982, *T*. *dryi* was imported into Mauritius^34^. In these islands both *T*. *erytreae* and *D*. *citri* coexisted as well as the African and Asian forms of HLB. However, only the classical biological control of *T*. *erytreae* with *T*. *dryi* was successful^34,35^. The lack of alternative hosts for *T*. *erytreae*, the presence of alternative hosts for *T*. *dryi* and the absence of hyperparasitoids were considered key aspects for the establishment of *T*. *dryi* and the successful control of *T*. *erytreae* populations^36–39^. Whether *T*. *dryi* would find these conditions in mainland Europe its unknown. However, *T*. *dryi* is highly specific. The parasitoid did not parasitize and develop in any of the eleven alternative host species that were offered in host-specificity tests^40^.

In this study, we propose a classical biological control programme to introduce the main parasitoids of *T*. *erytreae* from its area of origin into Europe. We first identified the parasitoid complex of *T*. *erytreae* in several areas of South Africa using morphological and molecular characterisation. We then determined several aspects of the biology of the main parasitoids: sex ratio, longevity and hyperparasitism. Implications of using *T*. *dryi* in classical biological control programmes of *T*. *erytreae* are discussed.

## Results

### Insect survey

The Western Cape was the only province in South Africa where neither *T*. *erytreae* nor its symptoms were recorded (see supplementary material Table S1). Leaves of trees from 17 out of the 65 sampled sites in the other provinces had the characteristic open gall-like structures, indicating the presence of *T*. *erytreae*. Live nymphs were collected from five out of the 17 sites and parasitoids were recorded from four sites.

### Parasitoid emergence and species abundance

**Figure 1:**
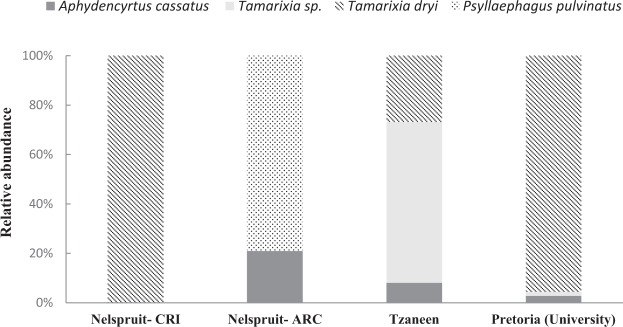
Relative abundance of *Trioza erytreae* parasitoids collected from individual parasitized nymphs at four sites in South Africa in 2017.

A total of 580 parasitized *T*. *erytreae* individuals were collected during the survey. From these samples 334 parasitoids belonging to five species emerged. The parasitoids in the remaining 246 psyllids failed to develop. The parasitoid complex was composed of at least five species whose relative abundance varied with sampling site (Fig. 1). *Tamarixia dryi* was the most abundant primary parasitoid in Pretoria and Nelspruit (>95% of the emerged parasitoids). This parasitoid species was also present in Tzaneen. On the other hand, the primary parasitoids *P*. *pulvinatus* (79%) and *Tamarixia* sp. (65%) were the most abundant species in Nelspruit and Tzaneen, respectively. The most abundant hyperparasitoid was *Aphidencyrtus cassatus* Annecke (Hymenoptera: Encyrtidae), which was recovered in Nelspruit, Tzaneen and Pretoria. One specimen of an *Aphanogmus* sp. (Hymenoptera: Ceraphronidae) was recorded in Tzaneen.

Parasitism rates ranged between 0.72 ± 0.12 in Pretoria and 0.41 ± 0.21 in Nelspruit (Table 1). Parasitoid emergence ranged between 0.57 ± 0.04 and 0.66 ± 0.19. Hyperparasitism rates ranged between 0 and 0.09.

**Table 1:** Parasitism and hyperparasitism rates of *Trioza erytreae* in four locations from South Africa.

Date	Location	Parasitism assessment			
		No. of nymphs examined	Parasitism rate [Mean ± EE (*n*)]	Parasitoid emergence [Mean ± EE (*n*)]	Hyperparasitism rate [Mean ± EE (*n*)]
9/28/2017	Nelspruit- CRI*	72	0.45 ± 0.19 (5)	0.64 ± 0.09 (5)	0 (5)
09/29/2017	Nelspruit- ARC*	266	0.41 ± 0.21 (4)	0.66 ± 0.19 (4)	0.09 ± 0.09 (4)
10/05/2017	Tzaneen	742	0.42 ± 0.07 (3)	0.57 ± 0.004 (3)	0.08 ± 0.04 (3)
10/05/2017	Pretoria (University)	142	0.72 ± 0.12 (5)	0.65 ± 0.12 (5)	0.02 ± 0.003(5)

^*^CRI: Citrus Research International (Nelspruit).

^*^ARC: Agricultural Research Council (Nelspruit).

### DNA barcoding of *Tamarixia* and *Trioza* specimens

**Figure 2:**
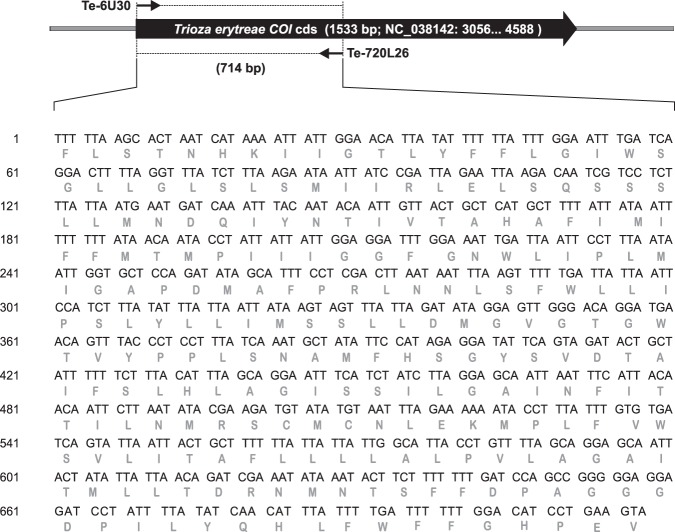
Nucleotide sequence of COI barcode fragment for *Tamarixia dryi* and *T*. sp generated in the present work. Deduced amino acid (aa) sequence of the corresponding polypeptide is shown under each triplet. Nucleotide changes and non-conserved aa in the sequence of of *T*. sp COI fragment are represented in boldface and in black boxes, respectively. The coding region of *Tamarixia* spp COI gene and standard primers position used for the amplification of the barcode fragment −652 bp without including the sequence of the standard primers– are shown for schematic purposes.

The sequences were submitted to the GenBank public sequence repository, with the following accession numbers for *T*. *dryi* (MK293946-MK293954), *Tamarixia* sp. (MK302489-MK302491) and *T*. *erytreae* (MK285548-MK285560). In a BLAST search, the COI barcode sequence obtained using the specific primers for *T*. *erytreae* showed 100% homology with the South African accessions KY754590 (TeSA7) and KY754594 (TeSA1) identified by Khamis *et al*.^41^ as *T*. *erytreae*, confirming that all specimens collected at Pretoria, Nelspruit and Tzaneen corresponded indeed to these species (see Supplementary Material Fig. S1). The COI barcode fragment sequenced from *T*. *dryi* and a new species of *Tamarixia* collected at Pretoria, Nelspruit and Tzaneen, shared 86–91% of identity to COI barcode fragment accessions from other *Tamarixia* species available in GenBank [*Tamarixia radiata* (Waterston)(GQ912272), *Tamarixia drukyulensis* (Yefremova and Yegorenkova) (KY986293) and *Tamarixia triozae* (Burks) (GQ912288)] (Fig. 2). The new species of *Tamarixia* collected at Tzaneen showed a higher identity (90%) to *T*. *dryi* sequences than to *T*. *radiata*, *T*. *drukyulensis* and *T*. *triozae* (87, 88 and 86%, respectively).

**Figure 3:**
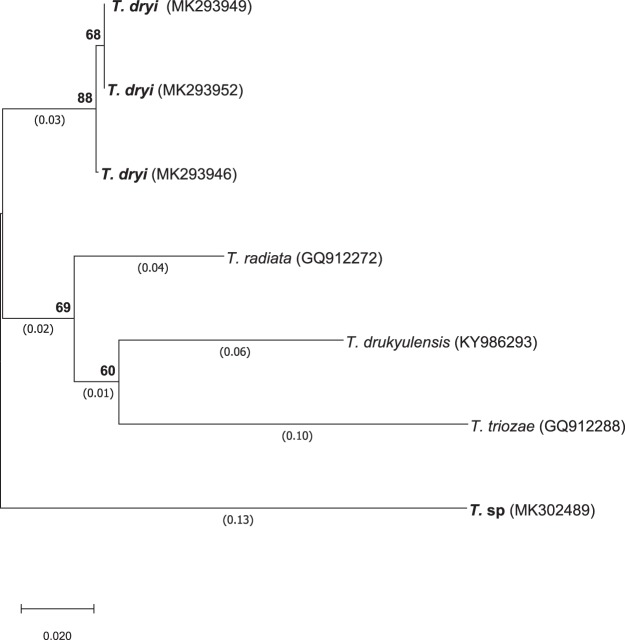
Rooted phylogenetic analysis showing the evolutionary relationships between *Tamarixia* species, based on DNA sequences of COI barcode fragment. The analysis involved seven nucleotide sequences including those of *Tamarixia dryi* and one *T*. sp generated in this study, and three closest sequences retrieved from GenBank [*T*. *radiata* (GQ912272), *T*. *drukyulensi*s (KY986293) and *T*. *triozae* (GQ812288)].

The phylogenetic tree of these *Tamarixia* species was paraphyletic with two distinct branches (Fig. 3). The first branch separated into two clusters. The first cluster grouped together the COI barcode sequences obtained in this work from the *T*. *dryi* specimens, while the second cluster hosted the *T*. *radiata*, *T*. *drukyulensis* and *T*. *triozae* GenBank accessions included in the analysis. The COI barcode sequence from the new *Tamarixia* sp. branched separately from the rest of the *Tamarixia* species included in this analysis.

### Seasonal trend of the parasitoid complex of *T*. *erytreae*

**Figure 4:**
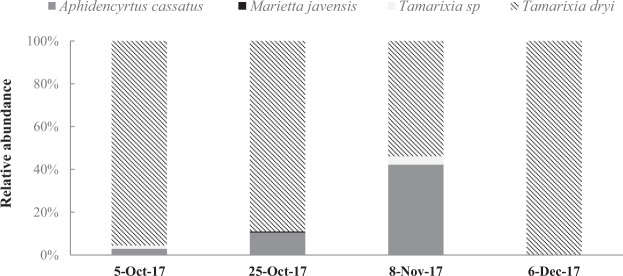
Relative abundance of *Trioza erytreae* parasitoids in a citrus orchard from the University of Pretoria (Pretoria, South Africa) throughout the spring of 2017.

The most abundant parasitoid was *T*. *dryi*, followed by its hyperparasitoid *A*. *cassatus*, but their relative abundance showed different trends (Fig. 4). While the relative abundance of *T*. *dryi* decreased over the five weeks, the number of hyperparasitoids increased during the same period. The primary parasitoid *Tamarixia* sp. was recorded during the first and third sampling periods, and the hyperparasitoid *Marietta javensis* (Howard) (Hymenoptera: Aphelinidae) was found only at the second sampling date.

### Effect of host size on the secondary sex ratio of *T*. *dryi* and *Tamarixia* sp

**Figure 5:**
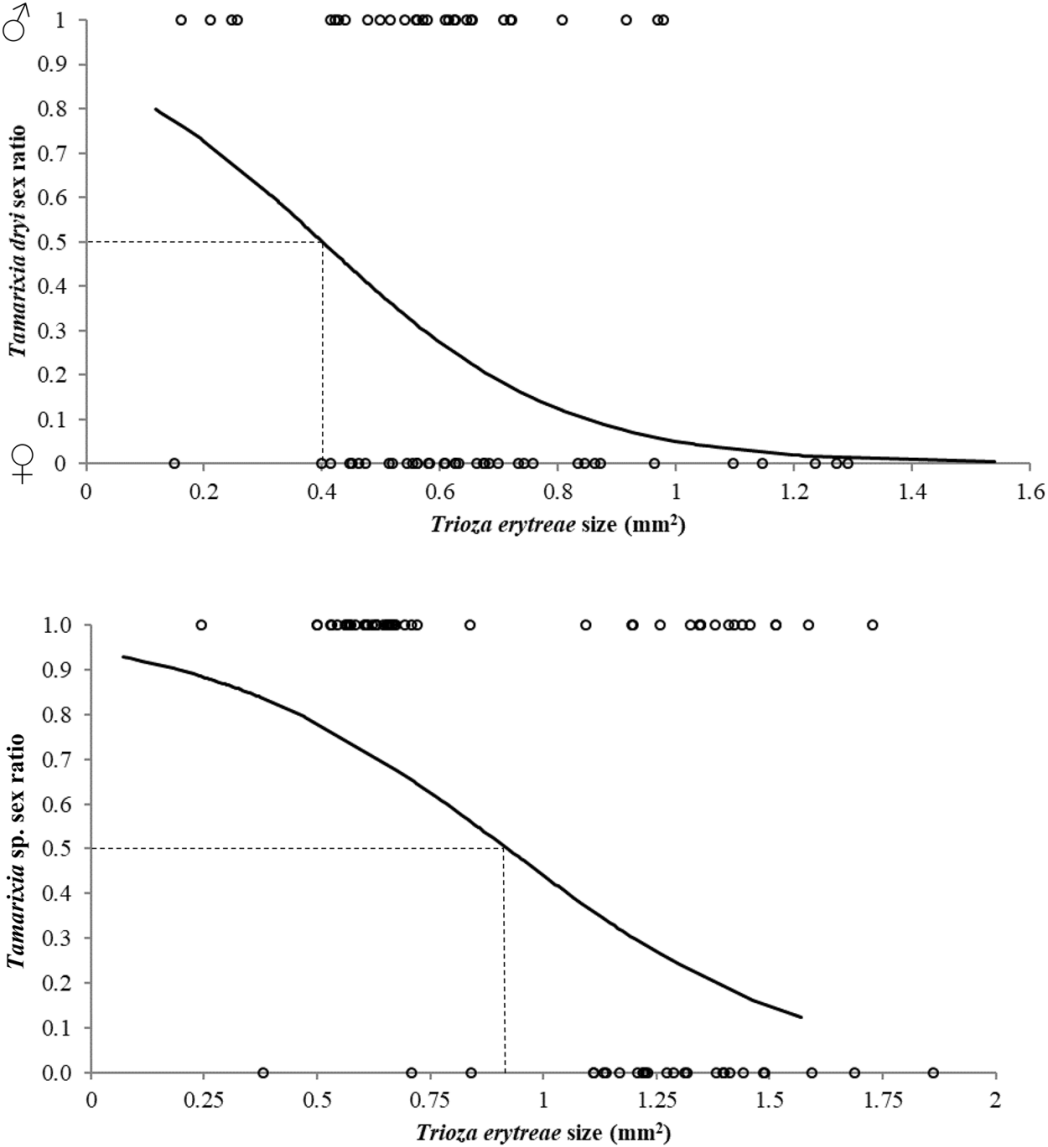
Effect of *Trioza erytreae* size on the secondary sex ratio of *Tamarixia dryi* (**A**) and *Tamarixia* sp (**B**). Sex ratio turns female-biased around 0.4 mm^2^ in *T*. *dryi* and 0.9 mm^2^ in *Tamarixia* sp.

The secondary sex ratio of the primary parasitoids *T*. *dryi* and *Tamarixia* sp. depended on *T*. *erytreae* size. In both species, females emerged from larger-sized hosts than males (*T*. *dryi*: *F* = −3.34; df = 1, 86; *P* < 0.001; *Tamarixia* sp.*: F* = −3.99; df = 1, 78; *P* < 0.001) (Fig. 5A,B). Sex ratio in *T*. *dryi* turned female-biased around 0.40 mm^2^ and in *Tamarixia* sp. at around 0.90 mm^2^.

### Effect of host size on *A*. *cassatus* emergence

**Figure 6:**
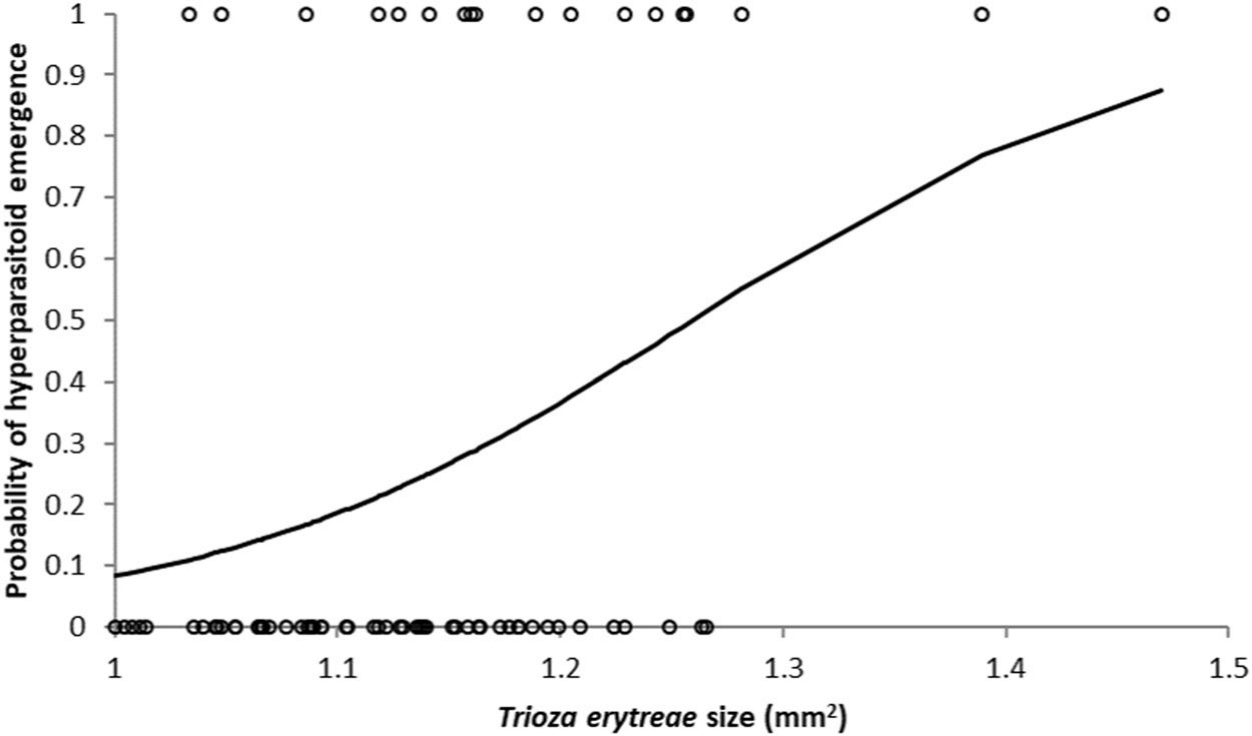
Effect of *Trioza erytreae* size on the probability that an individual of *Syrphophagus cassatus* emerged from the nymph.

Hyperparasitism also depended on the size of *T*. *erytreae* individuals. The hyperparasitoid *A*. *cassatus* tended to emerge from large hosts (*F* = 3.144; df = 1, 80; *P* = 0.002) (Fig. 6). Hyperparasitism rates became higher than 50% when hosts were larger than 1.65 mm^2^.

### Longevity of *T*. *dryi* and its hyperparasitoid *A*. *cassatus*

**Figure 7:**
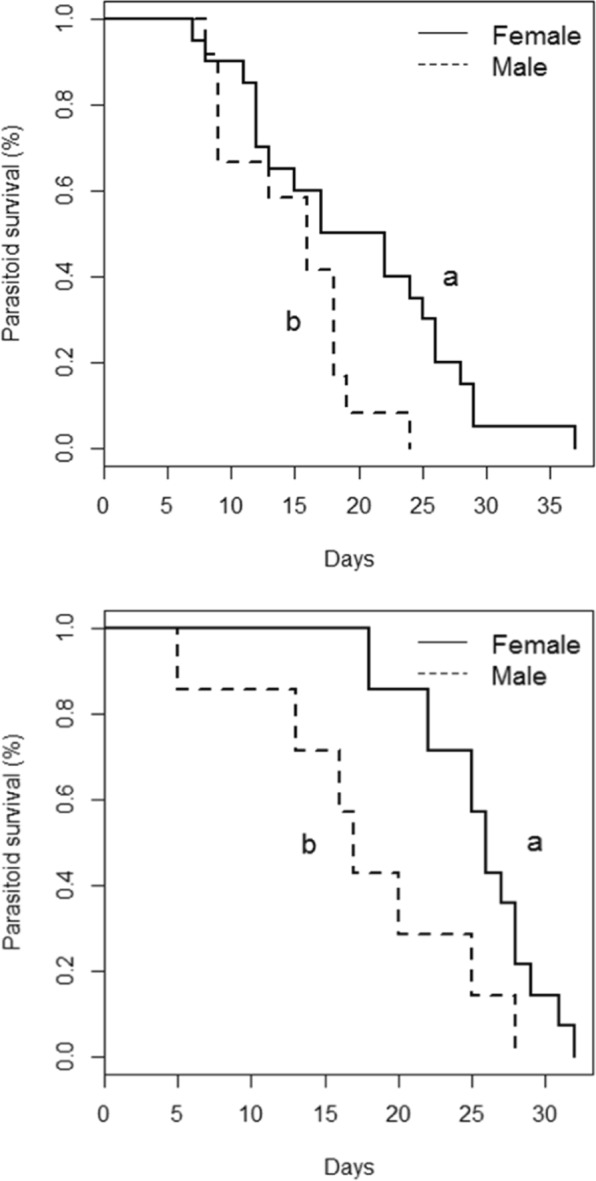
Cumulative survival function of *Tamarixia dryi* (**A**) and its hyperparasitoid *Syrphophagus cassatus* (**B**) for both sexes.

Survival of *T. dryi* differed between sexes (χ^2^_1_ = 4.29; *P* = 0.038) (Fig. 7). Females lived 19.6 ± 1.86 days on average and males 14.75 ± 1.47 days. Survival of *A. cassatus* was also higher for females than for males (25.5 ± 1.14 and 17.71 ± 2.89 days, respectively) (χ^2^_1_ = 5.16; *P* = 0.023). When females of both species were compared, no significant differences were found with respect to their longevity (χ^2^_1_ = 4.10; *P* = 0.21).

## Discussion

*Trioza erytreae* was highly parasitized by several species of hymenopteran parasitoids. Parasitism rates were high in all the sampled areas in spring and ranged between 0.40 and 0.70. These rates are similar to those reported by van der Merwe^42^ (1923) and Catling^43^ in South Africa and Swaziland, respectively, and Tamesse *et al*.^44^ in Cameroon. Therefore, as demonstrated by Catling in the 1960s, parasitoids are important biotic regulators of *T*. *erytreae* in those areas where insecticides are not sprayed in South Africa, e.g. abandoned and experimental orchards and public gardens^29,45^. This result reinforces the suggestion of introducing exotic parasitoids to those areas where *T*. *erytreae* has arrived and where effective native parasitoids are absent. This is the case of Maderia (Portugal), the Canary Islands (Spain) and, more recently, mainland Europe^15^.

Among the three species of primary parasitoids of *T*. *erytreae*, *T*. *dryi* was the most effective and abundant, as has been previously demonstrated by other studies in South Africa and Swaziland^30,43^. Its relative abundance was higher than 90% in two sites. Similar values were obtained by Catling^43^ in South Africa. Another primary parasitoid of *T*. *erytreae*, *P*. *pulvinatus*, was only present in Nelspruit-ARC, where it was the most abundant species (80%). The high abundance of *P*. *pulvinatus* at this site may be due to the absence of *T*. *dryi*. Although *P*. *pulvinatus* parasitizes younger nymphs than *T*. *dryi*, its developmental time is longer than that of *T*. *dryi*^30^. This reason might partially explain why *T*. *dryi* tends to be more abundant where *T*. *erytreae* is present at low population densities.

A new parasitoid species from the genus *Tamarixia* was recorded in Tzaneen and Pretoria. It was the most abundant species in Tzaneen (70%), coexisting with *T*. *dryi* and the hyperparasitoid *A*. *cassatus*. In Pretoria, it was recorded sporadically. This new species could be the same species named as “*Tetrastichus* sp. n” in Western and Eastern Africa and classified as a primary parasitoid (Aubert 1986). In other studies in Cameroon, Zimbabwe and Malawi, an unknown “*Tetrastichus* sp.” was also found, but it was recorded as a hyperparasitoid^30,34,44^. The high abundance of *Tamarixia* sp. and the fact that we never observed the pupae or larvae of any *Tamarixia* species attacked by other larvae suggest that it is a primary parasitoid. The molecular analysis confirmed that this new species has not been reported yet in the database Genbank and it is closely related to *T*. *dryi*.

The sex ratio of *T*. *dryi* and *Tamarixia* sp. turned female biased when the size of *T*. *erytreae* was greater than 0.40 and 0.90 mm^2^, respectively. Many species of solitary parasitoids lay male eggs on small hosts and female eggs on large hosts^46,47^. This host-size-dependent sex ratio is presumed to be advantageous because females gain a greater benefit from the resulting increase in body size than do males^46,48^. According to Waage^49^, this occurs mostly in idiobionts, which paralyze the host, because in koinobiont parasitoids, hosts continue growing and its size at oviposition is a less reliable predictor of the resources that offspring will have available for development^50,51^. In our case, although *T*. *dryi* is a koinobiont, host size increases only slightly after parasitism (personal observations). Therefore, host size could be a reliable predictor of the resources when *T*. *dryi* recognizes and assesses the size of *T*. *erytreae* individuals. These results are important for mass-rearing the parasitoids and maximizing the production of females^52^.

Hyperparasitism rates were low and ranged between zero and 0.1 at the four sites. In Pretoria, where the seasonal trend of the parasitoids was determined in spring, hyperparasitism also reached a maximum of 0.1. These values were higher than the ones obtained by Catling and Anneke in the Letaba District [Limpopo (then Transvaal province) South Africa] from 1965 to 1967, although hyperparasitism was thought to be of little apparent significance, later on Mc Daniel and Moran^30^ and Anneke and Moran^53^ determined that it was the main factor in the increase of *T*. *erytreae* population levels in citrus. Among the hyperparasitoids, *A*. *cassatus* was the most abundant and widely distributed. *Aphidencyrtus cassatus* is considered the most abundant hyperparasitoid of *T*. *erytreae* and it attacks, at least, the two primary parasitoids *T*. *dryi* and *P*. *pulvinatus*^43,44,54^. Moreover, *A*. *cassatus* has been observed host feeding in the thoracic region of the primary parasitoid host, which sometimes killed the host^30^. This mortality and the high temperatures reached at the end of spring could have caused the high mortality rates of the primary parasitoids observed in Pretoria.

Two traits of the biology of *A*. *cassatus* could explain the negative impact that this hyperparasitoid has on *T*. *dryi*. Firstly, the probability that the hyperparasitoid *A*. *cassatus* emerged from *T*. *erytreae* nymphs increased with the size of the nymph. Since *T*. *dryi* females develop in larger nymphs than males, *A*. *cassatus* may affect the secondary sex ratio of *T*. *dryi* and more males will emerge. Secondly, females of the hyperparasitoid *A*. *cassatus* lived for more than 30 days when they had access to carbohydrates, and their longevity was similar to that of *T*. *dryi*. Therefore, the hyperparasitoid is capable of surviving long periods of host scarcity or, at least, as long as the primary parasitoid. Although hyperaparasitoids can regulate herbivore populations by stabilising host-parasitoid interactions^55–57^, from the point of view of classical biological control, the accidental introduction of *A*. *cassatus* could impair the establishment of *T*. *dryi*^58^. Therefore, great care should be taken to exclude this parasitoid when importing *T*. *dryi* to Europe, for example introducing only adult parasitoids, establishing isolines and following quarantine procedures before the release^59,60^.

## Materials and Methods

### Insect survey

Sampling took place in citrus producing areas in four provinces, Gauteng, Limpopo, Mpumalanga and Western Cape, of South Africa. A total of 65 citrus orchards, 5 public parks and 60 private properties were examined from September 21st to December 9th of 2017. At all sites, the sampling date, the number of trees sampled, the variety of the trees and the presence of *T*. *erytreae* or its visual symptoms and parasitoids were recorded (see supplementary material Table S1). Visual symptoms of *T*. *erytreae* on citrus, in contrast to those of *Diaphorina citri*, consist of pit galls protruding from the upper surface of the leaves, chlorosis and leave twisting^15,61^.

### Parasitoid identification, relative abundance and parasitism rates

From those areas and trees where *T*. *erytreae* was collected, the psyllids were transported to the laboratory to identify potential parasitoids, determine their relative abundance and parasitism rates in each location. From each tree, 3 to 20 leaves infested by *T*. *erytreae* were collected and transported in enclosed individualized plastic bags to the laboratory. Due to the scarcity of *T*. *erytreae* in some of the orchards, the number of sampled trees and leaves was variable (see supplementary material Table S1). Once in the laboratory, the number of live psyllids and psyllids suitable for parasitism (2^nd^-to the 5^th^-instar nymph) and parasitized psyllids was recorded using a stereomicroscope. In order to identify all the parasitoids and calculate the rate of emerging parasitoids, psyllid nymphs were placed individually in 1 ml microtubes closed with cotton wool. Afterwards, the microtubes tubes were kept in an incubator (Labcon^TM^ 2 LTGC 20, Laboratory Marketing Services cc, South Africa) under controlled conditions (12 L: 12 Dh, 25 °C, 60–70% RH) and checked daily until parasitoids emerged. Once emerged, their sex was determined and the identification at species level was carried out using the key of Tamesse^62^. The morphological identifications of *T*. *erytreae* and *Tamarixia* parasitoids were confirmed by David Ouvrard (Natural History Museum, London) and Roger Burks (University of California, Riverside), respectively. Moreover, voucher specimens of all the parasitoid species collected during this study were deposited at the University of California, Riverside and labeled with the database number UCRC_ENT from 00517324 to 00517336.

In order to calculate the parasitism rate, each tree was used as a sampling unit because *T*. *erytreae*, as other psyllids, has an aggregative distribution pattern^63–65^.

### DNA extraction, PCR and sequencing of barcode fragment

The morphological identifications were verified with molecular identifications using cytochrome c oxidase subunit 1 (COI) sequences of *T*. *erytreae* and of the parasitoids *T*. *dryi* and *Tamarixia* sp. The insects were collected at three different locations, Nelspruit, Pretoria, and Tzaneen, in South Africa. DNA was extracted from individual insects using a salting out method^66^ adapted from Monzó *et al*.^67^. Polymerase chain reaction (PCR) was performed to amplify the mitochondrial COI gene. Standard primers LCO1490 (5′-GGTCAACAAATCATAAAGATATTGG-3′) and HCO2198 (5′-TAAACTTCA GGGTGACCAAAAAATCA-3′)^68^ were used for the amplification of a 708-bp fragment of the COI gene of *Tamarixia* species, while Te-6U30 (5′-ATTTTTAAGCACTAATCATAAAATTATTGG-3′) and Te-720L26 (5′-TATACTTCAGGATGTCCAAAAAATCA-3′) specific primers were used to amplify a COI barcode fragment of a 714-bp fragment of the COI of *T*. *erytreae*. PCR was carried out in a total reaction volume of 20 µl containing 1X Reaction Buffer (Mg free), 1625 µM MgCl2, 250 µM of all four dNTPs, 0.25 µM of each primer, 1 U of DNA polymerase (1 U/µl, Biotools), and 1 µl of DNA in a thermal cycler (Eppendorf Mastercycler). Reactions were cycled as follow: initial denaturation at 95 °C for 120 seconds (s); 40 cycles of 94 °C for 60 s, 45 °C for *Tamarixia* or 54 °C for *T*. *erytreae* for 60 s and 72 °C for 90 s; and a final extension at 72 °C for 600 s. Amplified PCR products were resolved in a 1.2% agarose gel and successfully amplified barcode fragments were cleaned up using the UltraClean PCR Clean-up DNA Purification Kit (MO BIO Laboratories Inc., Carlsbad - California, USA). Amplified barcode fragments were bidirectional sequenced using both the forward and reverse PCR primers. Sanger-sequencing was performed by capillary electrophoresis using a 3130XL Genetic Analyzer (Applied Biosystems, Carlsbad - California, USA), at the Sequencing Service of the IBMCP (Valencia, Spain). Sequences were analysed and trimmed to remove primer sequences, using Sequencer DNA Sequence Analysis Software (Gene Codes Corporation, Ann Arbor – Michigan, USA). Forward and reverse high-quality reads obtained for each individual were assembled into consensus sequences and submitted to the GeneBank public sequence repository.

### Sequence data and phylogenetic analysis

The phylogenetic analysis of the two species of *Tamarixia* was carried out using the COI barcode sequences obtained in this work together with those already included in the GenBank: *T*. *radiata* (Waterston), *T*. *drukyulensis* Yefremova and Yegorenkov and *T*. *trioza* (Burks). Consensus sequences corresponding to the amplified COI barcode fragment of the *Tamarixia* parasitoids were first used as query to BLASTN against the non-redundant nucleotide collection of the GenBank database (http://blast.ncbi.nlm.nih.gov/Blast.cgi), optimising for highly similar sequences. The barcode based taxonomic assignment at the genus level was set at 94% identity over a 90% sequence overlap. Multiple alignments of consensus sequences and closest sequence identities were done using Clustal Omega software^69^. The phylogenetic analysis was carried out in MEGA X^70^, using the Neighbor-Joining method^71^. The reliability of the tree pattern was evaluated using a bootstrap test with 10000 replicates^72^, and the evolution distances, given as units of the number of base substitution per sites, were computed using the Maximum Composite Likelihood method^73^. The rate variation among sites was modelled with a gamma distribution (shape parameter = 1).

### Seasonal trend of the parasitoid complex of *T*. *erytreae*

The seasonal trend of *T*. *erytreae* and its parasitoids was studied in an infested lemon orchard located at the University of Pretoria Experimental Farm (25°44′51.1″S 28°15′31.2″E). The orchard was ~10 years old, and the trees sampled were not treated with pesticidesduring the sampling period. From October to December, five infested leaves from five trees were sampled every two weeks. Samples were processed following the same methodology described above until parasitoids emerged and were identified.

### Effect of host size on secondary sex ratio and hyperparasitism of parasitoids

The effects of host size on the secondary sex ratio of *T*. *dryi* and *Tamarixia* sp. and on hyperparasitism were analyzed. After parasitoids emerged they were identified and their sex was determined. The psyllid nymphs have an oval shape and host size was determined by calculating the area of an ellipse by multiplying r_1_ × r_2_ × π (r_1_: major radius, r_2_: minor radius). Both sex and hyperparasitism ratios were analyzed using generalized linear models assuming binomial errors. The assumed error structure was assessed by a heterogeneity factor equal to the residual deviance divided by the residual degrees of freedom. If an over- or an under dispersion was detected, we re-evaluated the significance of the explanatory variables using an *F*-test after rescaling the statistical model by a Pearson’s *χ*2 divided by the residual degrees of freedom. All data analyses were performed with the R freeware statistical package (Version 1.0.143)^74^.

### Longevity of *Tamarixia dryi* and its hyperparasitoid *Aphidencyrtus cassatus*

*Tamarixia dryi* and *A*. *cassatus* longevity was recorded. From the individual nymph sampled at the University of Pretoria Experimental Farm, a total of 20 females and 12 males of *T*. *dryi* as well as 14 females and 7 males of *A*. *cassatus* were selected. Individual parasitoids were placed singly in 1 ml microtubes tubes closed with cotton wool and 1 M sucrose drop renewed every two days. The microtubes were kept under controlled conditions in an incubator (Labcon 2 LTGC 20, Laboratory Marketing Services CC, Roodepoort, South Africa) at 12L: 12D, 25 ± 2 °C and 60–70% RH, and the survival of the insects was checked daily. The Cox regression model was used to determine differences between sexes within the same species and between species. Analyses were carried out using the R freeware statistical package (Version 1.0.143)^74^.

### Ethical approval

This article does not contain any studies with human participants performed by any of the authors. All applicable international, national, and/or institutional guidelines for the care and use of animals were followed.

## Supplementary information

Supplementary information
