# Supplementary material for: Classical biological control of the African citrus psyllid Trioza erytreae, a major threat to the European citrus industry
Source: Sci Rep. 2019 Jul 1;9:9440. doi: 10.1038/s41598-019-45294-w (PMC6603031; doi:10.1038/s41598-019-45294-w)
Supplement: Supplementary file 1 — Supplementary information [file 41598_2019_45294_MOESM1_ESM.pdf]

Supplementary Information

Title: Classical biological control of the African citrus psylla *Trioza erytreae*, a major threat to the European citrus industry

Author list: J. Pérez-Rodríguez, K. Krüger, M. Pérez-Hedo, O. Ruíz-Rivera, A. Urbaneja, A. Tena.

Supplementary Table S1. Sampling dates and sites, number and variety of sampled trees per site and symptoms and presence of *T. erytreae.* Asterisks are used when *T. erytreae* was parasitized.

Sampling date	Locality	Province	Coordinates	Type of orchard	Rutacea Host	No. trees sampled	*Trioza* symptons	Presence of *Trioza*
21/09/2017	Citrusdal	Western Cape	32°36'27.3"S 18°56'37.8"E	Commercial	*Citrus sinensis*	50	No	No
21/09/2017	Citrusdal	Western Cape	32°36'31.6"S 18°56'39.9"E	Commercial	*Citrus reticulata*	50	No	No
21/09/2017	Citrusdal	Western Cape	32°36'23.3"S 18°56'51.7"E	Commercial	*Citrus sinensis*	50	No	No
21/09/2017	Citrusdal	Western Cape	32°36'37.2"S 18°56'32.1"E	Commercial	*Citrus limon*	50	No	No
21/09/2017	Citrusdal	Western Cape	32°35'43.2"S 19°00'50.2"E	Private garden	*Citrus sinensis*	2	No	No
21/09/2017	Citrusdal	Western Cape	32°51'18.0"S 19°05'55.8"E	Commercial	*Citrus sinensis*	50	No	No
21/09/2017	Citrusdal	Western Cape	32°51'19.0"S 19°05'40.4"E	Commercial	*Citrus sinensis*	50	No	No
21/09/2017	Citrusdal	Western Cape	32°36'37.3"S 19°00'01.2"E	Organic commercial	*Citrus limon*	50	No	No
21/09/2017	Citrusdal	Western Cape	32°36'27.2"S 18°59'55.5"E	Organic commercial	*Citrus limon*	50	No	No
21/09/2017	Citrusdal	Western Cape	32°36'42.4"S 19°00'05.5"E	Organic commercial	*Citrus reticulata*	50	No	No
22/09/2017	Citrusdal	Western Cape	32°37'03.5"S 18°57'20.6"E	Organic commercial	*Citrus reticulata*	10	No	No
22/09/2017	Citrusdal	Western Cape	32°37'03.5"S 18°57'20.6"E	Private garden	*Citrus sinensis*	4	No	No
22/09/2017	Citrusdal	Western Cape	32°37'03.5"S 18°57'20.6"E	Private garden	*Citrus limon*	2	No	No
22/09/2017	Citrusdal	Western Cape	32°21'23.4"S 18°55'52.5"E	Organic commercial	*Citrus sinensis*	50	No	No
22/09/2017	Citrusdal	Western Cape	32°21'28.7"S 18°55'56.2"E	Organic commercial	*Citrus sinensis*	50	No	No
22/09/2017	Stellenbosch	Western Cape	33°49'21.8"S 18°55'48.1"E	Private garden	*Citrus sinensis*	100	No	No
22/09/2017	Stellenbosch	Western Cape	33°56'11.0"S 18°51'56.3"E	Private garden	*Citrus sinensis*	5	No	No
26/09/2017	Nelspruit	Mpumalanga	25°27'09.0"S 30°58'06.4"E	Organic commercial	*Citrus sinensis*	50	No	No
26/09/2017	Nelspruit	Mpumalanga	25°27'07.9"S 30°58'16.1"E	Organic commercial	*Citrus limon*	50	No	No
27/09/2017	Nelspruit	Mpumalanga	25°23'04.3"S 30°32'33.8"E	Nursery	*Citrus sinensis*	20	Yes	No
27/09/2017	Nelspruit	Mpumalanga	25°23'06.7"S 30°32'34.1"E	Commercial	*Citrus sinensis*	20	No	No
27/09/2017	Nelspruit	Mpumalanga	25°22'44.2"S 30°31'50.7"E	Organic commercial	*Citrus limon*	50	No	No
27/09/2017	Nelspruit	Mpumalanga	25°22'44.2"S 30°31'50.7"E	Abandoned orchard	*Citrus reticulata*	10	No	No
27/09/2017	Nelspruit	Mpumalanga	25°22'35.5"S 30°32'30.8"E	Private garden	*Citrus limon*	1	No	No
27/09/2017	Nelspruit	Mpumalanga	25°22'49.1"S 30°34'03.0"E	Private garden	*Citrus limon*	1	No	No
27/09/2017	Nelspruit	Mpumalanga	25°22'35.7"S 30°32'33.0"E	Commercial	*Citrus limon*	1	No	No
27/09/2017	Nelspruit	Mpumalanga	25°26'42.8"S 30°57'57.4"E	Public garden	*Citrus aurantium*	0	No	No
28/09/2017	Nelspruit	Mpumalanga	25°27'31.5"S 31°02'46.5"E	Commercial	*Citrus sinensis*	0	Yes	No
28/09/2017	Nelspruit	Mpumalanga	25°27'12.2"S 31°01'58.2"E	Abandoned orchard	*Citrus sinensis*	20	No	No
28/09/2017	Nelspruit	Mpumalanga	25°27'30.2"S 31°02'35.0"E	Abandoned orchard	*Citrus limon*	20	No	No
28/09/2017	Nelspruit	Mpumalanga	25°27'58.2"S 31°02'32.4"E	Commercial	*Citrus limon*	50	No	No
28/09/2017	Nelspruit	Mpumalanga	25°28'48.4"S 30°59'38.2"E	Experimental farm	*Citrus limon*	30	Yes	Yes*

29/09/2017	Nelspruit	Mpumalanga	25°27'07.7"S 30°58'09.5"E	Nursery	*Citrus sinensis*	50	No	No
29/09/2017	Nelspruit	Mpumalanga	25°27'07.4"S 30°58'15.9"E	Assay	*Citrus limon*	50	Yes	Yes*
29/09/2017	Nelspruit	Mpumalanga	25°28'21.9"S 30°59'29.8"E	Public garden	*Citrus aurantium*	10	Yes	No
29/09/2017	Nelspruit	Mpumalanga	25°28'45.8"S 30°59'38.0"E	colony	*Citrus sinensis*	20	No	No
03/10/2017	Letsitele	Limpopo	23°52'01.0"S 30°23'20.0"E	Privat garden	*Murraya exotica*	1	No	No
03/10/2017	Letsitele	Limpopo	23°52'07.0"S 30°23'29.6"E	Privat garden	*Murraya exotica*	3	No	No
03/10/2017	Letsitele	Limpopo	23°51'44.7"S 30°23'01.9"E	Commercial	*Citrus sinensis*	50	No	No
03/10/2017	Tzaneen	Limpopo	23°55'11.8"S 30°13'58.4"E	Private garden	*Citrus limon*	3	No	No
03/10/2017	Tzaneen	Limpopo	23°50'24.0"S 30°18'07.2"E	Commercial	*Citrus sinensis*	150	No	No
04/10/2017	Tzaneen	Limpopo	23°55'11.8"S 30°13'58.4"E	Organic commercial	*Citrus sinensis*	150	Yes	No
04/10/2017	Tzaneen	Limpopo	23°54'42.1"S 30°13'28.0"E	Organic commercial	*Citrus sinensis*	150	Yes	No
04/10/2017	Tzaneen	Limpopo	23°53'14.7"S 30°19'44.8"E	Organic commercial	*Citrus sinensis*	150	Yes	No
04/10/2017	Tzaneen	Limpopo	23°55'06.3"S 30°13'59.5"E	Organic commercial	*Citrus sinensis*	150	Yes	No
04/10/2017	Tzaneen	Limpopo	23°55'07.9"S 30°13'55.4"E	Organic commercial	*Citrus sinensis*	150	Yes	No
04/10/2017	Nkowankowa-C	Limpopo	23°53'24.4"S 30°19'40.7"E	Private garden	*Citrus limon*	2	No	No
04/10/2017	Nkowankowa-C	Limpopo	23°53'27.9"S 30°19'39.6"E	Private garden	*Citrus limon*	3	Yes	No
04/10/2017	Nkowankowa-C	Limpopo	23°53'25.6"S 30°19'42.1"E	Private garden	*Citrus limon*	1	No	No
04/10/2017	Nkowankowa-C	Limpopo	23°53'19.3"S 30°19'43.8"E	Private garden	*Citrus limon*	2	No	No
04/10/2017	Nkowankowa-C	Limpopo	23°53'31.0"S 30°19'45.3"E	Private garden	*Citrus limon*	1	Yes	No
05/10/2017	Tzaneen	Limpopo	23°50'13.7"S 30°09'37.8"E	Private garden	*Citrus limon/reticulata*	5	Yes	Yes*
05/10/2017	Tzaneen	Limpopo	23°47'56.8"S 30°26'11.0"E	Private garden	*Citrus limon*	5	No	No
05/10/2017	Tzaneen	Limpopo	23°47'54.1"S 30°26'07.9"E	Private garden	*Citrus limon*	50	No	No
05/10/2017	Pretoria	Gauteng	25°44'52.8"S 28°15'32.1"E	Experimental farm	*Citrus limon*	10	No	No
05/10/2017	Pretoria	Gauteng	25°44'52.1"S 28°15'33.6"E	Experimental farm	*Citrus limon*	25	Yes	Yes*
09/10/2017	Pretoria	Gauteng	25°45'40.6"S 28°14'12.5"E	Private garden	*Citrus sinensis*	4	No	No
09/10/2017	Pretoria	Gauteng		Private garden	*Citrus limon*	3	Yes	Yes
09/10/2017	Pretoria	Gauteng		Private garden	*Murraya exotica*			
09/10/2017	Pretoria	Gauteng	25°44'21.4"S 28°16'24.7"E	Public garden	*Citrus aurantium*	10	No	No

10/10/2017	Pretoria	Gauteng		Private garden	*Citrus limon*	5	Yes	No
20/10/2017	Pretoria	Gauteng	25°45'30.5"S 28°14'31.4"E	Public garden	*Citrus sinensis*	3	No	No
01/11/2017	Pretoria	Gauteng		Private garden	*Citrus limon*	1	Yes	No
17/11/2017	Pretoria	Gauteng	25°49'55.6"S 27°53'20.3"E	Public garden	*Citrus sinensis*	5	No	No
09/12/2017	Pretoria	Gauteng		Private garden	*Citrus limon*	6	Yes	Yes*

Supplementary Figure S1. Nucleotide sequence of COI barcode fragment for *Trioza erytreae* generated in the present work. Deduced amino acid (aa) sequence of the corresponding polypeptide is shown under each triplet. The coding region (cds) of *T. erytreae* COI gene and specific primers position used for the amplification of the barcode fragment –714 bp including the sequence primers– are shown for schematic purposes. Coordenates of COI cds are given with respect to the mitochondrial nucleotide sequence of *T. erytreae* (GeneBank accesession number: NC_038142).

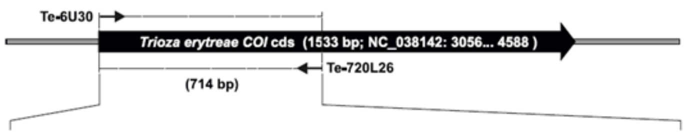


```
1     TTT TTA AGC ACT AAT CAT AAA ATT ATT GGA ACA TTA TAT TTT TTA TTT GGA ATT TGA TCA
       F   L   S   T   N   H   K   I   I   G   T   L   Y   F   F   L   G   I   W   S
61    GGA CTT TTA GGT TTA TCT TTA AGA ATA ATT ATC CGA TTA GAA TTA AGA CAA TCG TCC TCT
       G   L   L   G   L   S   L   S   M   I   I   R   L   E   L   S   Q   S   S   S
121   TTA TTA ATG AAT GAT CAA ATT TAC AAT ACA ATT GTT ACT GCT CAT GCT TTT ATT ATA ATT
       L   L   M   N   D   Q   I   Y   N   T   I   V   T   A   H   A   F   I   M   I
181   TTT TTT ATA ACA ATA CCT ATT ATT ATT GGA GGA TTT GGA AAT TGA TTA ATT CCT TTA ATA
       F   F   M   T   M   P   I   I   I   G   G   F   G   N   W   L   I   P   L   M
241   ATT GGT GCT CCA GAT ATA GCA TTT CCT CGA CTT AAT AAT TTA AGT TTT TGA TTA TTA ATT
       I   G   A   P   D   M   A   F   P   R   L   N   N   L   S   F   W   L   L   I
301   CCA TCT TTA TAT TTA TTA ATT ATA AGT AGT TTA TTA GAT ATA GGA GTT GGG ACA GGA TGA
       P   S   L   Y   L   L   I   M   S   S   L   L   D   M   G   V   G   T   G   W
361   ACA GTT TAC CCT CCT TTA TCA AAT GCT ATA TTC CAT AGA GGA TAT TCA GTA GAT ACT GCT
       T   V   Y   P   P   L   S   N   A   M   F   H   S   G   Y   S   V   D   T   A
421   ATT TTT TCT TTA CAT TTA GCA GGA ATT TCA TCT ATC TTA GGA GCA ATT AAT TTC ATT ACA
       I   F   S   L   H   L   A   G   I   S   S   I   L   G   A   I   N   F   I   T
481   ACA ATT CTT AAT ATA CGA AGA TGT ATA TGT AAT TTA GAA AAA ATA CCT TTA TTT GTG TGA
       T   I   L   N   M   R   S   C   M   C   N   L   E   K   M   P   L   F   V   W
541   TCA GTA TTA ATT ACT GCT TTT TTA TTA TTA TTG GCA TTA CCT GTT TTA GCA GGA GCA ATT
       S   V   L   I   T   A   F   L   L   L   L   A   L   P   V   L   A   G   A   I
601   ACT ATA TTA TTA ACA GAT CGA AAT ATA AAT ACT TCT TTT TTT GAT CCA GCC GGG GGA GGA
       T   M   L   L   T   D   R   N   M   N   T   S   F   F   D   P   A   G   G   G
661   GAT CCT ATT TTA TAT CAA CAT TTA TTT TGA TTT TTT GGA CAT CCT GAA GTA
       D   P   I   L   Y   Q   H   L   F   W   F   F   G   H   P   E   V
```
